# Bioactive Peptides Derived from Edible Insects: Effects on Human Health and Possible Applications in Dentistry

**DOI:** 10.3390/nu15214611

**Published:** 2023-10-30

**Authors:** Gianmaria Fabrizio Ferrazzano, Francesca D’Ambrosio, Sara Caruso, Roberto Gatto, Silvia Caruso

**Affiliations:** 1 UNESCO Chair in Health Education and Sustainable Development, Paediatric Dentistry Section, University of Naples “Federico II”, 80138 Naples, Italy; gianmariafabrizio@yahoo.it; 2 Department of Laboratory and Infectious Diseases Sciences, Fondazione Policlinico Universitario A. Gemelli IRCCS, 00168 Rome, Italy; 3 Department of Life, Health and Environmental Sciences, University of L’Aquila, 67100 L’Aquila, Italy; saracaruso2704@gmail.com (S.C.); roberto.gatto@univaq.it (R.G.); silvia.caruso@univaq.it (S.C.)

**Keywords:** edible insects, nutrition, bioactive peptides, antihypertensive, antioxidant, anti-inflammatory, antimicrobial, metabolic conditions, dentistry, antibiotic resistance

## Abstract

Novel foods, including edible insects, are emerging because of their nutritional characteristics and low environmental impacts and could represent a valid alternative source of food in a more sustainable way. Edible insects have been shown to have beneficial effects on human health. Insect-derived bioactive peptides exert antihypertensive, antioxidant, anti-inflammatory, and antimicrobial properties and have protective effects against common metabolic conditions. In this review, the roles of edible insects in human health are reported, and the possible applications of these peptides in clinical practice are discussed. A special mention is given to the role of antimicrobial peptides and their potential applications in controlling infections in orthodontic procedures. In this context, insects’ antimicrobial peptides might represent a potential tool to face the onset of infective endocarditis, with a low chance to develop resistances, and could be manipulated and optimized to replace common antibiotics used in clinical practice so far. Although some safety concerns must be taken into consideration, and the isolation and production of insect-derived proteins are far from easy, edible insects represent an interesting source of peptides, with beneficial effects that may be, in the future, integrated into clinical and orthodontic practice.

## 1. Introduction

Historically, insects have been part of the diets of many cultures in Asia, Africa, and Latin America [1]. Moreover, it has been estimated that edible insects are part of the diets of over two billion people worldwide [2].

Edible insects have been recently identified as “novel food” by the European Commission under Regulation (EU) 2015/2883, together with foods or ingredients that are not usual in human diets. Novel foods have been recently emerging for both their nutritional potentials and low impacts on the environment. To date, about 2000 insect species are documented as edible, including beetles, caterpillars, wasps, bees, ants, crickets, etc. [3].

In 2013, Rumpold and Schluter analyzed the nutrient compositions of 236 edible insects [4]. Moreover, the International Network of Food Data Systems (INFooDS) included edible insects’ nutritional values in their database.

Edible insects are rich in proteins, unsaturated fatty acids (including polyunsaturated fatty acids like omega-3), and micronutrients like copper, iron, magnesium, phosphorous, zinc, riboflavin, biotin, and, in some cases, folic acid [4]. For example, house crickets (*Acheta domesticus*) and grasshoppers have high protein contents (61% dry basis), followed by dragonflies (55% dry basis) [5].

Furthermore, edible insects such as crickets and yellow mealworms (*Tenebrio molitor*) have protein contents comparable to those of pork and beef [6].

Considering the nutritional values, these foods could be a valid alternative to traditional diets supplements. This is particularly relevant to face the constantly growing demand for food necessitated by the increasing global population. Being a sustainable source of proteins, edible insects could mitigate the problem of malnutrition in developing and poor countries [7].

Farmed insects require fewer resources to produce the same amounts of protein than domesticated animals, reduce environmental impacts in terms of greenhouse gases and ammonia emissions, and allow us to save greater amounts of water [8]. An interesting study of Zielinska et al. showed the capability of two insect species (*Tenebrio molitor* and *Zophobas morio*) to consume and degrade polystyrene, without side effects on insects and probably on humans after entomophagy. This shed a light on the potential roles of insects in plastic degradation, which impacts environmental pollution [9].

An interesting definition defines functional foods as “foods formulated so that they contain substances or live microorganisms that have a possible health-enhancing or disease-preventing value, and at a concentration that is both safe and sufficiently high to achieve the intended benefit” [10].

This definition expands the concept introduced by the Food and Agriculture Organization of the United Nations (FAO), according to which novel foods could be beneficial to human health. In fact, many novel foods, rich in bioactive compounds, have been shown to have therapeutic potentials in a wide variety of pathologic conditions. Among the extracts authorized as novel foods for their uses in food supplements, those more studied in the literature are plant-origin ones [11]. Nonetheless, unusual novel foods (including insects) are given more attention due to their potentials in human health.

In this review, we discuss the potential effects of bioactive peptides derived from edible insects on human health and their possible applications in the management of different pathologic conditions. We also give a general overview of the methodologies employed in the purification and identification of bioactive peptides. Finally, we speculate on, for the first time, the introduction of antimicrobial peptides derived from insects in orthodontic practice.

## 2. Purification and Isolation of Bioactive Peptides

Isolating and identifying bioactive peptides from the whole insect require several steps, which we will discuss below.

The first steps are protein isolation and hydrolysis. Protein hydrolysis is crucial to obtain biologically active peptides from the total amounts of insects’ proteins. For this reason, several proteases may be involved, including alcalase and flavourzyme [12,13]. Alcalase is probably the most effective and commonly used in research, combining extensive hydrolysis (the enzyme shows both exo- and endo-proteinase activities) with a high protein content [14]. Moreover, some studies used a combination of proteases to simulate gastrointestinal digestion and evaluate the stability and bioavailability of the target proteins. For example, Vercruysse et al. used pepsin, trypsin, and α-chymostrypsin, which are physiologically involved in the digestion of proteins [15]. Mudd et al., instead, obtained gastrointestinally digested peptides, employing pepsin, pancreatin, and bile salts [16]. Eventually, the peptides’ isolation may be optimized by pre-treatments, for example, using a PH-shift method before adding proteases [17,18].

After obtaining protein hydrolysates, peptides must be purified with biochemical assays. The most commonly used method is chromatography, but non-chromatographic techniques may also be chosen. Gel filtration/liquid chromatography allows for fractionating peptides and separating them according to molecular size, through a gel or liquid matrix [19,20]. Usually, a first purification occurs before chromatography, by means of ultrafiltration. This method is based on the filtration of a hydrolysate through a system of membranes, which separates the different fractions of a sample based on their molecular weights. This step may determine the fraction with the best biological activity [13,20]. Eventually, the fraction that displays the strongest activity can be further purified with reversed-phase high-performance liquid chromatography (RP-HPLC) [18].

The identification step allows targeting of the peptide or peptides of interest, starting from the fraction with the highest biological activity. For the identification, mass spectrometry (MS) techniques are widely accepted, since peptides may be targeted with high specificity, even in complex samples. Different methods of mass spectrometry are used, starting from MS/MS, liquid chromatography–mass spectrometry (LC-MS/MS), ESI–tandem mass spectrometry (ESI-MS/MS), electrospray ionization–mass spectrometry (ESI-MS), or matrix-assisted laser desorption/ionization–MS (MALDI-MS) [14,21,22].

Once the peptide/peptides of interest have been identified, specific assays can be performed to evaluate the biological activity of the molecules.

Many studies also perform a molecular docking, an in silico structure-based method which enables us to predict the possible ligand–target interactions and to estimate the binding free energy. Although molecular docking finds application mainly in the pharmacological field, a variety of docking algorithms are available nowadays [23,24]. In this context, docking is used to investigate the interactions between the insects’ derived peptides and the target enzyme/protein to provide a better insight to the mechanism of action of the peptides and/or to narrow the search field to target proteins. Wu et al., through the molecular docking software Discovery Studio 2.1, analyzed the angiotensin-I-converting enzyme (ACE) inhibition by a tripeptide derived from silkworm pupae, attributed to strong hydrogen bonds with the S1 and S2 pockets [20]. Moreover, Pattarayingsakul et al. used the molecular docking software AutoDock V.4 to select the peptides that exhibited the more favorable predicted binding energy values [19].

## 3. Edible Insects and Human Health

Research suggests that insects’ proteins, after enzymatic proteolysis, may be a good source of bioactive peptides [5]. The positive impact of bioactive peptides on body functions and, thus, on human health is well acknowledged [25]. Indeed, bioactive peptides exert antioxidant, anticancer, antithrombotic, antihypertensive, antiobesity, anti-inflammatory, opioid, mineral binding, immunomodulatory, antiaging, and antimicrobial effects, with high organ specificity and low toxicity [26].

### 3.1. Antihypertensive Effects

High blood pressure is a major risk factor for cardiovascular diseases [27]. Globally, more than one billion people are considered hypertensive and hypertension is responsible for up to ten million deaths worldwide [28]. Interestingly, insect-derived bioactive peptides show antihypertensive properties. The best-characterized method of action consists in the inhibition of ACE. This enzyme is part of the renin–angiotensin–aldosterone system (RAAS), playing a pivotal role in blood pressure regulation. Indeed, the inhibition of ACE prevents the conversion of angiotensin I to angiotensin II and, for this reason, ACE inhibitors are commonly used as pharmacological therapies for patients with hypertension [29]. Many peptides show high affinity in binding ACE because of their C-terminal amino acid sequences [30]. For example, the peptide YETGNGIK, derived from locusts, includes amino acids like isoleucine and lysine, which bind ACE with high affinity, exerting a strong inhibition (half-maximal inhibitory concentration, IC_50_: 3.25 μg/mL) [31].

In an in vivo study of Yuan et al., a silk fibroin hydrolysate (SFH) exhibited blood pressure lowering and an improvement in endothelial functions in models of spontaneously hypertensive rats (SHRs) [32]. SFH, derived from alcalase hydrolysis with a hydrolysis degree of 17%, inhibits ACE [12], strictly correlated with the development of hypertension and atherosclerosis [33].

Vercruysse et al. studied the antihypertensive properties of cotton leafworm (*Spodoptera littoralis*) both in vitro and in vivo. The authors obtained a hydrolysate using pepsin, trypsin, and α-chymostrypsin to simulate the human gastrointestinal digestion process. Afterwards, the ACE inhibitory tripeptide Ala-Val-Phe was isolated by chromatography and RP-HPLC. This peptide showed good ACE inhibition activity in vitro, with an IC_50_ of 2123 μM [15]. Vercruysse et al. also evaluated the stability and activity of the tripeptide Ala-Val-Phe in vivo. The tripeptide was partially hydrolyzed by mucosal peptidases into the peptide Val-Phe, which showed a stronger ACE inhibition. Moreover, after a single oral administration (5 mg/kg body weight), the dipeptide induced a significant decrease in blood pressure in models of SHRs [34].

Also, the peptide GNPWM (603.7 Da; IC_50_ = 21.70 μM) and four modified peptides purified from silkworm pupae (*Bombyx mori*) directly inhibit ACE and could be beneficial as functional drugs or foods to treat hypertension [18]. Another study of Wu et al. investigated the antihypertensive activity of peptides derived from silkworm pupae. Peptides were hydrolyzed with three gastrointestinal peptidases (pepsin, trypsin, and α-chymostrypsin) to simulate digestion. Eventually, a novel tripeptide (Ala-Ser-Leu) was identified by mass spectrometry and was a competitive inhibitor of ACE, as suggested by Lineweaver–Burk plots [20].

Besides having protective effects against hypertension, bioactive peptides derived from yellow mealworms (*Tenebrio molitor*) may exert antithrombotic activities. The antihypertensive effects were reported by Dai et al. In their study, the novel peptide Tyr-Ala-Asn was purified and identified by RP-HPLC and mass spectrometry from a yellow mealworm larva hydrolysate. This tripeptide significantly decreased the blood pressure in in vivo models of SHsR [21]. Chen et al., instead, investigated the antithrombotic potential of enzymatic hydrolysates derived from *Tenebrio molitor* larvae, obtained after a treatment with pepsin and trypsin. After separation and purification processes, two peptides were identified (SLVDAIGMGP and AGFAGDDAPR), showing a strong antithrombotic activity, with values of thrombin inhibition activity of 40.87% and 65.61% at 8.0 mg/mL, respectively [35].

Thus, research suggests the important role of bioactive peptides derived from insects in the regulation of blood pressure and their protective cardiovascular effects.

### 3.2. Antidiabetes, Antiobesity, and Hepatoprotective Effects

Over the past few decades, the prevalence of diabetes has risen significantly, representing a burden in worldwide healthcare [36]. Also, obesity remains the main risk factor for the development of diabetes [37]. Surprisingly, bioactive peptides derived from many insects show antidiabetic properties both in vitro and in vivo. The main targets of those peptides investigated so far are α-amylase, α-glucosidase, and dipeptidyl peptidase IV (DDP-IV) inhibition. Both α-glucosidase and α-amylase enzyme inhibitors can suppress peaks of postprandial glucose, counteracting hyperglycemia, as previously demonstrated by studies conducted on botanical compounds including polyphenols [38]. Alternatively, DDP-IV is responsible for the degradation of glucagon-like peptide-1 (GLP-1), decreasing its insulinotropic effect and worsening diabetes conditions [39].

Bioactive peptides derived from edible crickets (*Gryllodes sigillatus*) show both antihypertensive and antidiabetic properties targeting ACE, α-glucosidase, and α-amylase.

This multiple protective effect is particularly interesting since diabetes and hypertension are two closely related conditions, sharing common mechanisms, such as upregulation of the renin–angiotensin–aldosterone system, oxidative stress, and inflammation [40].

The hydrolysated peptides LPDQWDWR and APPDGGFWEWGD from *Tenebrio molitor*, instead, show good inhibitory effects on DDP-IV, with IC_50_ values of 0.15 and 1.03 mg/mL, respectively [17]. An in vivo study of Han et al. showed the hypoglycemic effects of the tripeptides Gly-Glu-Tyr (GEY) and Gly-Tyr-Gly (GYG), isolated from the silk peptide E5K6 in a model of steptozotocin (STZ)-induced diabetic mice in a dose-dependent manner [41]. Indeed, the hydrolysated E5K6 protein exerts many protective functions, lowering not only glucose levels but also total cholesterol and low-density lipoprotein (LDL) plasma levels in vivo [42].

Bioactive peptides isolated from edible insects have also shown antiobesity properties. The prevalence of obesity has increased globally in recent decades. This heterogeneous condition also causes concern due to the associated comorbidities (cardiovascular conditions, type 2 diabetes, etc.), causing millions of deaths worldwide [43].

Chemically synthetized fractions derived from the peptide sequence of edible insects target lipases, thus reducing lipidic absorption and accumulation. The fractions IIAPPER from *Gryllodes sigillatur* and AIGVGAIER from *Schistocerca gregaria* showed high lipase inhibition, with an IC_50_ of 49.44 and 49.95 μg/mL, respectively [31].

Obesity is often characterized by a hormonal dysregulation and, in this context, the hormone leptin has a pivotal role, leading to reduced satiety, increased body mass, and overconsumption of nutrients [44]. However, the feeling of satiety also depends on the chemical composition of foods [45]. Being rich in proteins, edible insects have highly satiating properties as discussed in a recent work of Skotnicka et al. [46]. In this study, pancakes were prepared with flour derived from *Tenebrio molitor*, *Alphitobius domesticus*, and *Acheta domesticus* in three different proportions. The physico-chemical composition of the pancakes was characterized and the satiating potential of this food in a population of seventy-one volunteers was assessed using a visual analogue scale (VAS). As a result, the products richer in protein best suppressed hunger and promoted satiety, supporting the idea of introducing edible insects in antiobesity diets [46].

As well as antiobesity properties, edible insects also have hepatoprotective effects. Fan et al. investigated the potential role of AGL9, the enzymatic hydrolysate of the beetle larva of *Allomyrina dichotoma*, in moderating hepatic steatosis in a non-alcoholic fatty liver disease (NAFLD) murine model. After administration of AGL9 for five weeks, mice showed normalized levels of alanine aminotransferase, aspartate aminotransferase, triglyceride, total cholesterol, high-density lipoprotein, very low-density lipoprotein (VLDL), adiponectin, and leptin. Indeed, AGL9 inhibits the AMPK/Nrf2 signaling pathway, thus decreasing lipid accumulation and the production of proinflammatory mediators [47].

Collectively, all these findings highlight the protective properties of bioactive peptides derived from edible insects against complex metabolic conditions that represent a burden on health nowadays.

### 3.3. Antioxidant and Anti-Inflammatory Effects

The antioxidant effects of insects’ bioactive peptides have been widely investigated, although the precise mechanisms are still poorly understood. Many edible insects showed antioxidant potential, including crickets, mealworms, and locusts [22].

One of the most powerful antioxidant peptides isolated so far is FDPFPK, derived from *Gryllodes sigillatur*. The peptides were obtained by in vitro digestion and separated by gel filtration chromatography. Eventually, proteins were identified with mass spectrometry methods and antioxidant potential was assessed with both 2,2′-azino-bis(3-ethylbenzothiazoline-6-sulfonic acid) (ABTS) and 2,2-diphenyl-1-picrylhydrazyl (DPPH) assays. As a result, the peptide FDPFPK showed a high antiradical activity with IC_50_ values of 0.08 and 0.35 mg/mL for ABTS and DPPH, respectively [22].

Moreover, a study of Mudd at al. showed the antioxidant effects of simulated gastrointestinally digested (SGD) peptides from *Gryllodes sigillatus*, both in vitro and in vivo, on a model of *Caenorhabditis elegans.* SGD peptides increased *Caenorhabditis elegans* lifespan under oxidative stress conditions, reducing reactive oxygen species (ROS) levels. Also, SGD peptides increased gene glutathione S-transferase alpha 4 (gsta-4) expression through the activation of the epidermal growth factor (EGF) pathway [16].

Larvae and pupae of Asian weaver ants (*Oecophylla smaragdina*) exert both antihypertensive and antioxidants effects. In particular, the fraction CTKKHKPNC showed radical-scavenging activity (ABTS assay: IC_50_, 38.4 μg/mL) and antioxidant activity (DPPH assay: 48.2 μg/mL) [19].

Studies of Zhang et al. and Li et al. highlighted the antioxidant properties of peptides derived from houseflies’ larvae (*Musca domestica*). Zhang et al. compared the antioxidant activities of hydrolysates obtained either with alcalase or neutral proteinase. In both cases, hydrolysates showed up to 50% superoxide anion radical-scavenging activity [13]. Li et al. evaluated the amino acidic composition of housefly larvae. Interestingly, according to the authors, the presence of hydrophobic and aromatic amino acids in the water extract of horseflies may contribute to the radical-scavenging activity [48].

Hydrolysates from silkworm pupae (*Bombyx mori*) have been investigated for their antioxidant potential [14,49]. FKGPACA and SVLGTGC fractions showed radical-scavenging activity in vitro (ABTS assay: IC50, 0.312 mM and 0.181 mM, respectively) [49]. Furthermore, SWFVTPF and NDVLFF fractions showed high superoxide dismutase (SOD) activity with values of 36.96 ± 7.31 and 30.43 ± 7.90% SO inhibition on a culture of HepG2 cells, and all peptide-treated cells also showed significantly high glutathione (GSH) levels [14].

Inflammation is one of the major risk factors for the progression of several chronic diseases, including cancer, diabetes, and cardiovascular disorders [50]. In addition to immune response cells, which are the main actors in the inflammatory process, several mediators play a pivotal role in inflammation. The enzymes belonging to the family of cyclooxygenase (COX) and lipoxygenase (LOX) are implicated in the production of prostaglandins and leukotrienes, respectively, from arachidonic acid [51]. Both eicosanoids can be powerful mediators of inflammation and, for this reason, COX and LOX enzymes are the target of many drugs for the treatment of inflammation [51,52].

Bioactive peptides identified from edible insects showed promising anti-inflammatory effects by inhibiting COX and LOX enzymes.

As well as displaying strong antioxidant properties, peptide fractions obtained from *Gryllodes sigillatus* were shown to have anti-inflammatory effects in vitro. These peptides are effective inhibitors of COX-2 (IC_50_: 0.26 µg/mL) and LOX (IC_50_: 0.13 µg/mL) as shown by a cyclooxygenase inhibitory activity assay and lipoxygenase inhibitory activity assay, respectively [22].

Tang et al. isolated thirteen non-peptide nitrogen-containing compounds from Chinese black ants (*Polyrhachis dives*) and tested their anti-inflammatory activity on murine macrophage cells (RAW264.7 cells) treated with lipopolysaccharides (LPSs). These compounds inhibited not only the production of tumor necrosis factor-α (TNF-α) and COX-1 but also Jak3 kinase activity and T lymphocytes, but no significant inhibition was detected for B-cells as shown by T- and B-lymphocyte proliferation assays [53].

Baek et al. obtained an aqueous fraction of *Lycorma delicatula* and tested the anti-inflammatory properties in LPS-treated RAW264.7 cells. The fraction inhibited nitric oxide production at a concentration of 300 μg/mL. Moreover, interleukin (IL)-13 induced by LPS and matrix metalloproteinase (MMP)-7, -9, -14, and -17 levels were significantly decreased [54].

Collectively, all these results support the idea that edible insects could ameliorate the severity of the inflammatory process, although the mechanisms of action must be better understood.

### 3.4. Insects’ Antimicrobial Peptides

Most living organisms, ranging from fungi to plants and animals, can produce antimicrobial peptides (AMPs). These small (15–30 amino acids) and positively charged peptides play a significant role in killing pathogens (bacteria, fungi, viruses, and some parasites) through the modulation of the host’s innate response and are particularly effective against biofilms. Interestingly, since the purification of cecropin, the first insect AMP from *Hyalophora cecropia* in 1980 [55], about 150 AMPs have been isolated from *Acalolepta luxuriosa, Apis mellifera*, *Bombyx mori, Galleria mellonella, Heterometrus spinifer,* and *Holotrichia diomphalia* [56,57].

According to their amino acidic components, insect AMPs can be classified in four groups: the α-helical peptides (e.g., cecropin and moricin), cysteine-rich peptides (e.g., insect defensin and drosomycin), proline-rich peptides (e.g., apidaecin, drosocin, and lebocin), and glycine-rich proteins (e.g., attacin and gloverin). Among them, defensins, cecropins, proline-rich peptides, and attacins are probably the most common insects’ AMPs [57].

Insect defensins are probably the best-characterized AMPs and are active in particular against Gram-positive bacteria, for example *Bacillus subtilis, Bacillus thuringiensis*, and *Staphylococcus aureus*, although antibacterial effects have also been shown on Gram-negatives such as *Escherichia coli* [58]. In a study of Kaneko et al., a defensin sequence was isolated from *Bombyx mori*. Interestingly, the expression of the defensin gene was stimulated by the infection of *E. coli* and *B. subtilis*, showing an implication of this AMP in the immune response against these bacteria [59].

Many cysteine-rich peptides with different names may be considered defensin-like proteins, including coprisins. In a study of Hwang et al., coprisin was isolated from a dung beetle (*Copris tripartitus*). In vitro studies showed an upregulation of coprisin genes after infection with bacteria, followed by strong antimicrobial activity against *S. aureus* and *E. coli* [60]. Moreover, coprisin shows a synergistic activity with common antibiotics like ampicillin and vancomycin against many bacteria (*Enterococcus faecium*, *Streptococcus mutans*, *E. coli* O-157, and *Pseudomonas aeruginosa*) and inhibits biofilm formation [61].

The ability of AMPs to inhibit biofilm formation deserves a special mention, since the matrix surrounding bacteria makes them more resistant to antibacterial treatments and responsible for a wide range of chronic diseases [62]. In addition to coprisin, many are further examples of AMPs involved in biofilm inhibition. Defensin-1, the recombinant form derived from European honeybee defensin, showed antibiofilm efficacy against *S. aureus*, *Streptococcus agalactiae*, and *P. aeruginosa* (minimum inhibitory concentration, MIC: 0.009–0.09 μM) [63], and melittin, which is the principal component of the venom of honeybees, showed high MIC values against *P. aeruginosa* and a synergic activity with antibiotics against *E. coli and Klebsiella pneumoniae* [64]. Notably, mauriporin derived from *Androctonus mauritanicus* and Mastoparan-1 derived from *Polybia paulista* are significantly effective against methicillin-resistant S. aureus (MRSA), with a MIC of 5–10 and 0.001–0.019 μM, respectively [65,66].

In recent years, the focus on the application of AMPs in clinical practice has increased notably because of their interesting characteristics, such as the broad spectrum of antimicrobial activity, the small size, and the low chance for development of resistances [67]. For these reasons, some insect-derived AMPs (i.e., ETD151, thanatin) have been optimized by genetic manipulation and have been tested in in vivo models with promising antibacterial properties [68,69,70]. These observations support the idea of extending the experimentation and study of these molecules to various fields of medicine, including dentistry.

## 4. Potential Applications of Edible Insects’ AMPs in Dentistry

Previous studies investigated the role of functional food in prevention and management of oral diseases. Notably, cyanobacteria and microalgae are receiving attention for the richness of high-value compounds (i.e., carotenoids, vitamins, and proteins) and for their beneficial effects on human health, being a source of anti-inflammatory and antioxidant molecules [71,72,73]. In the specific case of oral health, cyanobacteria and microalgae may find application as promising drugs in oral cancer prevention, oral submucosal fibrosis treatment, and in the management of chronic periodontitis [74,75,76,77]. Furthermore, as for insects, these functional foods have antimicrobial activity against microorganisms like *Herpes labialis*, *Streptococcus mutans*, and *Candida albicans*, representing a potential application in oral infection prevention [77].

In fact, one of the most important challenges that dentistry faces is the onset of oral infections, which may lead to serious clinical outcomes. The case of infective endocarditis (IE) is emblematic. Indeed, an antibiotic prophylaxis (AP) is recommended before orthodontic procedures in order to lower the risk of infection, especially in patients at high risk [78]. AP, that has been widely used in the past, is now controversial because of the emergence of multidrug-resistant strains of bacteria, mostly *Staphylococci*, *Streptococci*, and *P. aeruginosa* [79]. As discussed above, AMPs derived from edible insects display a broad spectrum of antimicrobial activities, both against Gram-positive and Gram-negative bacteria, representing a potential tool to treat oral infections with a low chance of developing resistances. For this reason, AMPs might be manipulated, optimized, and introduced into dental practice as substitutes for the common antibiotics used to date. Further studies may focus on the possible effects of the combined administration of AMPs derived from edible insects and molecules with antimicrobial activity extracted from other functional foods (i.e., cyanobacteria and microalgae) to investigate possible synergistic effects. The manipulation and transformation of edible insects deserve a special mention, allowing a better tolerance of insect intake and palatability. Previous studies investigating the role of functional foods (e.g., algae) in dentistry usually employed local gels or tablets [74,80,81]. Also, an interesting study of Rashad et al. synthesized silver nanoparticles against oral pathogens [82]. Silver nanoparticles are well documented as antibacterial, antifungal, and antiviral agents and are employed in different fields, including drug delivery and diagnosis [83]. In addition to the above-mentioned administration methods, novel drug delivery systems could be considered, including chewing gums or toothpastes for topical effects. Flours extracted from edible insects could be processed to make foods like pancakes, biscuits, or bread [46,84]. These foods based on insect flours may be better tolerated, even by pediatric patients.

Despite AMPs appearing to be promising candidates for the treatment of different infections and as an interesting tool to solve the problem of microbial resistance, there are many limitations for drug development. The process of induction, extraction, and optimization of these molecules is far from easy and requires expertise and has high costs, despite the high availability of insects.

## 5. Limits in the Application and Consumption of Edible Insects

An increase in insect consumption is expected in the next decade, with an annual rate of 20–30% [1]. This trend reflects the awareness of the benefits of entomophagy, including sustainability, nutritional properties, and beneficial effects on human health. However, the consumption of insects faces several limitations. First, there is a lack of legislation to regulate the production and commercialization of edible insects [5]. Another important topic is safety. In fact, like other foods derived from plants or animals, insects can accumulate pathogens or contain residues of pesticides and heavy metals from the ecosystem, so stringent controls need to be conducted on insects before being marketed [85]. Allergens also represent an important issue. According to databases (e.g., www.allergen.org, accessed on 20 June 2023), tropomyosin and arginine kinase are the most important allergens that have been identified in edible insects [86]. This represents a notable risk for allergy sufferers and unfortunately there are no effective methods that can completely eliminate allergenicity to date [86]. To consider edible insects as an alternative source of food, digestibility must be considered as well. According to several in vitro studies, which used a combination of gastric enzymes to simulate gastrointestinal digestion, the protein digestibility of edible insects is very high (between 80% and 90%) [87]. However, the enzymes of monogastric animals are not effective in digesting acid detergent fiber, including chitin [88]. Chitin is a long-chain polymer regularly found in the exoskeleton of insects [89], but studies showed that, despite the presence of chitinolytic enzymes in the human microbiome, its removal improved protein digestibility [90,91]. However, processing methods are available: gut extraction, different methods of killing (freezing or blanching), obtaining protein isolates, defatting, thermal processing (drying or cooking), and extrusion, that improve the digestion of edible insects [87].

Furthermore, edible insects are not widely accepted by a great part of the population and the term “food neophobia” has been introduced to describe the reluctance to try novel foods. This attitude is particularly associated with higher age, living alone, and a shorter education [92]. Interestingly, a study of Orkusz et al. showed that participants preferred to eat processed insect foods (i.e., bread, biscuits) rather than unprocessed whole insects [84]. Therefore, new mechanisms to improve the acceptability of insects should be applied, for example, transforming them into unrecognizable and more palatable forms. Finally, ethical considerations about the application of edible insects in medicine or nutrition must be considered. A growing number of studies underline consumers’ concerns about the importance of insects’ welfare. Criteria of welfare may be different from those of vertebrates, and it is still debated whether insects are sentients beings or not. Currently, there is a lack of research in the area of insects’ welfare, especially regarding species-specific needs, farming systems, and killing methods. However, in line with the law, insects, like other farmed animals, must not be manipulated in a way that inflicts unnecessary pain or harm on them [93,94].

## 6. Conclusions

Edible insects represent an important source of bioactive peptides, exerting notable effects on human health. The antihypertensive, antioxidant, anti-inflammatory, and antimicrobial properties along with the protective effects against metabolic conditions shed light on the possible application of these proteins in clinical practice of different fields of medicine, including dentistry.

More studies are required to evaluate the toxicity and the mechanisms of action of AMPs. Also, further efforts are necessary to investigate the application of insect-derived bioactive proteins in the therapeutic field, since edible insects may represent a sustainable and valid alternative for both nutrition and medicine.

## Data Availability

Not applicable.

## Abbreviations

International Network of Food Data Systems (INFooDS), the Food and Agriculture Organization of the United Nations (FAO), reversed-phase high-performance liquid chromatography (RP-HPLC), mass spectrometry (MS), liquid chromatography–mass spectrometry (LC-MS/MS), ESI–tandem mass spectrometry (ESI-MS/MS), electrospray ionization–mass spectrometry (ESI-MS), matrix-assisted laser desorption/ionization–MS (MALDI-MS), angiotensin-I-converting enzyme (ACE), renin–angiotensin–aldosterone system (RAAS), half-maximal inhibitory concentration (IC_50_), silk fibroin hydrolysate (SFH), spontaneously hypertensive rats (SHRs), dipeptidyl peptidase IV (DDP-IV), glucagon-like peptide-1 (GLP-1), steptozotocin (STZ), low-density lipoprotein (LDL), visual analogue scale (VAS), non-alcoholic fatty liver disease (NAFLD), very low-density lipoprotein (VLDL), 5′ AMP-activated protein kinase (AMPK), nuclear factor erythroid 2-related factor 2 (Nrf2), 2,2′-azino-bis(3-ethylbenzothiazoline-6-sulfonic acid) (ABTS), 2,2-diphenyl-1-picrylhydrazyl (DPPH), simulated gastrointestinally digested (SGD), reactive oxygen species (ROS), glutathione S-transferase alpha 4 (gsta-4), epidermal growth factor (EGF), superoxide dismutase (SOD), gluthatione (GSH), cyclooxygenase (COX), lipoxygenase (LOX), lipopolysaccharides (LPSs), tumor necrosis factor-α (TNF-α), interleukin (IL), matrix metalloproteinase (MMP), antimicrobial peptides (AMPs), methicillin-resistant S. aureus (MRSA), minimum inhibitory concentration (MIC), infective endocarditis (IE), antibiotic prophylaxis (AP).
